# mTOR, glycotoxins and the parallel universe

**DOI:** 10.18632/aging.101720

**Published:** 2018-12-12

**Authors:** Alan S. Green

**Affiliations:** 1 The Medical Practice of Dr. Alan S. Green, Little Neck, NY 11363, USA

**Keywords:** advance glycation end products, aging, rapamycin, food restriction

Mikhail Blagosklonny’s most recent paper, “Disease or not, aging is easily treatable” [1] presents a very good solution to a difficult issue. I have been following the Blagosklonny’s Koschi formula in which intermittent rapamycin is the cornerstone of treatment for the past three years, since age 73 with excellent results. I have also developed an anti-aging clinical practice for the past two years, treating over 200 patients. The treatment plan, also closely follows the 2017 Blagosklonny paper, “From rapalogs to anti-aging formula” [PMID: 28548953] in which intermittent oral rapamycin is the cornerstone of treatment. In the Blagosklonny July 2018 paper [PMID: 30100983] there are 4 references documenting epigenetic changes in mouse liver and mouse brain secondary to oral rapamycin. For people waiting for epigenetic studies to show rapamycin has anti-aging effects, these studies provide that evidence. Even before the first 2009 study showing oral rapamycin given to middle age mice prolonged lifespan, the Blagosklonny 2006 paper [PMID: 17012837] set forth the theory for anti-aging and predicted this result. However, the widespread treatment to slow aging by physicians has not happened. For most people anti-aging treatment is like, “Do-it-yourself Brain Surgery.”

Perhaps one reason why anti-aging treatment by physicians has not materialized, is we live in parallel universes. Different groups have their own reality. In the world of the clinical practice of classical medicine, mTOR does not exist. The universe described by Blagosklonny in a series of papers from 2006 to present setting forth the concepts of mTOR and hyperfunction, speeding cars without brakes and pre-disease remains in a parallel universe.

Recently, jumping into the parallel universe of classical medicine, I discovered a very exciting body of science, about a remarkable group of chemicals originally called “browning products”; AKA glycotoxins, also known by the acronym AGEs for advance glycation end products.

Browning products first appeared ages ago when man first discovered the chemistry of fire and food. Early man quickly learned that not only did fire preserve food; but it made animal products taste much better. In 1912, the French chemist Louis Camille Maillard, identified the specific chemical reaction and applied the name “browning products”. Anybody who has ever tasted fried bacon, smelled a pizza fresh from the oven, seen an oven roasted turkey or eaten grilled steak is very familiar with these “browning products”. These chemicals are extremely well known to cooks, to the entire food industry and to just about anybody who eats animal products that are broiled, grilled, fried or baked. However, until very recently they remained outside the universe of medicine.

Beginning in the mid 1970s, a group of researchers studying diabetes turned their attention to something called non-enzymatic glycation. This led to the discovery of HgA1c, glycated hemoglobin, which came to be very widely recognized as a measure of average blood glucose. Glycated hemoglobin was a post- translational non-enzymatic modification of hemoglobin by glucose which caused hemoglobin to lose the ability to function properly in carrying oxygen. This was just the tip of a very huge iceberg. It was soon recognized that glucose through non-enzymatic glycation reacted with the amino acids in the lens which destroyed the pristine clear quality of lens proteins and resulted in yellow and finally brown cataracts, the AGE food colors of roasted turkey. The researchers became more and more impressed with the toxic nature of these chemicals. They were so impressed they gave these chemicals the acronym AGE for advanced glycation end products and to emphasize their toxic role in age-related disease. They did notice the chemical similarity to Maillard’s “browning products” in cooking foods; so much so that an inside joke among the researchers was that “we are all slowly being cooked”. However, up until 1995, there was the still the very firm belief that “browning products” in foods were not absorbed and played no role in human disease. AGEs were considered a strictly endogenous problem. This all changed in 1995 when it was suddenly discovered that “browning products” in foods were absorbed and were in indeed the major source of AGEs in the body [2].

There are hundreds of different AGEs produced in foods when animal products are subjected to high dry heat. Some are harmless and inactive and some are very reactive oxidants, which generate free radicals. The body has excellent ability to defend against these toxins. AGEs are seized and then elimination by the kidneys. However, once AGE could be measured and quantified it was determined that the body could only eliminate about 5000-8000 KU of AGEs a day. Older persons have reduced kidney function and any persons with chronic kidney disease have a decreased ability to clear AGEs. The average intake of AGEs in standard Western diet is 12,000 to 20,000 KU. People on a standard Western diet frequently consume 2 to 3 times the level of AGEs the body can eliminate. AGEs that exceed the body’s ability to eliminate accumulate in the body. As they accumulate they act as chronic poisons and cause havoc. The health benefits of the Mediterranean diet and the Vegetarian diet over the standard Western diet appears to be due to the lesser amounts of AGEs in these diets.

As excess AGEs accumulate in the body and cause a litany of harmful effects. They act as oxidants and cause oxidant stress overwhelming the body’s natural antioxidant defenses. Excess AGEs attach to proteins and form cross-links, which make tissue like blood vessels, heart muscle and the lens stiff. They react with surface pattern receptors on tissues called RAGE and cause chronic inflammation. Excess AGEs react with proteins and histones causing random post translational modifications with all that entails. The net result all the harmful actions of excess AGEs initially is pre-disease and slow progression to what looks like chronic age-related disease and aging.

It is hard to find an age-related disease that AGEs are not involved; but any agent causing age-related disease will certainly be involved in neurodegeneration. In healthy young persons an intact blood-brain barrier excludes AGEs from the brain. However, in older persons, when the blood-brain barrier becomes leaky, excess AGEs can gain access to the brain and cause both cognitive decline and help promote Alzheimer’s disease. An in vitro study showed that AGEs modified beta amyloid seeds and accelerated the aggregation of soluble beta amyloid compared to non-AGE modified seeds. The same study showed that AD brains had 3-fold more AGEs than healthy, age-matched controls. The suggestion was the greater accumulation of AGEs in the brain promoted greater formation of amyloid aggregates [3]. As regards cognitive decline, another study showed that in elderly, the rate of cognitive decline over 5 years was directly related to the blood level of methyglyoxal at the start of the study [4]. Methyglyoxal is a highly reactive AGE directly related to amount of dietary intake of AGEs.

The research team at Mount Sinai School of Medicine, NYC, doing a large share of the research over the past 30 years regarding AGE, in two companion studies dealt a huge blow to the venerated dogma that feeding mice a food restricted diet is equivalent to caloric restriction [5,6]. Standard mouse chow is exposed to high heat for preservation. This has the effect of producing high levels of AGE in mouse chow comparable to the Standard Western Diet. In Cai, 2007 one group of mice was fed regular chow and the other group was fed mouse chow that was subjected to less heat and thereby had 50% less AGE. The group of low AGE fed mice had 15% increased median lifespan, decreased kidney disease characterized by less glomerulosclerosis, improved insulin sensitivity and improved other markers of metabolic health.

In the Cai, 2008 study, they specifically challenged the dogma of caloric restriction. They state in the Abstract: “Because reduction of food intake also decreases oxidant AGEs intake, we asked whether the beneficial effects of CR in mammals are related to restriction of oxidants (AGEs) or energy (CR).” They had 3 groups of mice: (a) standard chow, (b) standard chow, 40% food restriction, (c) 40% food restriction, AGE enriched food. The results were the mice fed 40% less standard chow had less kidney disease (glomerulosclerosis), less cardiac disease (cardiac fibrosis) increased maximum lifespan, 13% increased median survival compared to mice on same chow; but without 40% food restriction. The mice on 40% food restriction plus enhanced AGEs had no benefit. In fact, they did worse than the control mice in all categories. The 40% caloric restriction mice with enhanced AGEs had shorter life span, increased cardiac and renal fibrosis and increased metabolic markers associated with decreased lifespan. Other studies showed that reduced AGEs resulted in increased SIRT1; of special note because the classic difference between “CR” and oral rapamycin is unlike rapamycin, “CR” increases SIRT1 which is associated with improved survival. The AGE studies showed increased SIRT1 is result of decrease in AGEs.

Of course, oral rapamycin increases lifespan in mice without any change in food intake. Caloric restriction is known to reduce mTOR. Therefore, a reasonable interpretation of the above two studies is classic 40% food restriction in mice studies enjoys BOTH the benefits of reduction of mTOR plus the benefits of 40% reduction in AGEs.

The AGE Less diet is the name given to diet with reduced AGEs. The AGE Less diet involves no caloric restriction and no medication. It involves avoiding the very worst foods (fried bacon) a reduction in very high AGE foods and cooking with moist heat instead of dry heat. It is about using chemistry to produce less AGEs in foods instead of more AGEs. Anybody who is willing to substitute having a poached egg instead of a fried egg can rather easily adjust to an AGE Less diet.

The reason I am promoting the AGE Less diet is because I believe it will have excellent synergy with oral rapamycin. The classic studies involving food restriction show excellent synergy between caloric restriction which reduces mTOR when inadvertently combined with AGE reduction. Although the science of AGEs is very well documented with over 8000 papers listed in PubMed, it is little known to both health care professionals and the general public In an important regard, the science of AGEs shares a common characteristic with rapamycin; both have zero commercial value.

I consider the combined use of oral intermittent rapamycin and the AGE Less diet to have the best potential to treat aging. However, I do not expect either to become very popular as they both suffer from the same fatal flaw. Rapamycin is a generic drug. The AGE Less diet is just cooking the same foods with low moist heat. However, until a super expensive new anti-aging drug comes along that can make Big Pharma 50 billion dollars a year, generic rapamycin and the AGE Less diet will do quite well.
